# Association between neutrophil percentage-to-albumin ratio and prevalence peripheral artery disease in U.S. adults: a cross-sectional study from the NHANES

**DOI:** 10.1186/s12872-025-05001-2

**Published:** 2025-07-21

**Authors:** Shou-yi Wang, Qianq Zhou, A-bing Li, Yang-kai Zhao

**Affiliations:** 1 Department of Interventional Vascular Surgery, The Third Affiliated Hospital of Shanghai University (Wenzhou People’s Hospital), Wenzhou, 325000 PR China; 2 Department of Orthopedics, Wenzhou Hospital of Integrated Traditional Chinese and Western Medicine, 75 Jinxiu Road, Wenzhou, Zhejiang 325000 China; 3 Department of Orthopedics, Ningbo Yinzhou Second Hospital, Ningbo Zhejiang, Ningbo, Zhejiang 315100 China

**Keywords:** Ankle-brachial index, Peripheral artery disease, Neutrophil-percentage-to-albumin ratio, National health and nutrition examination survey

## Abstract

**Background:**

Inflammation plays a key role in the progression of Peripheral arterial disease (PAD). The objective of this research is to explore the possible link between the neutrophil-to-albumin ratio (NPAR) and the occurrence of PAD.

**Methods:**

In this cross-sectional study of the National Health and Nutrition Examination Survey (NHANES) 199–2004, data from 5,470 participants were analyzed. The Ankle-brachial index (ABI) was obtained by dividing the mean systolic blood pressure in the ankle by the mean blood pressure in the arm. PAD was characterized by an ABI value of less than 0.9 in either leg. Patients diagnosed with PAD and those without, all of whom had detailed NPAR data from NHANES. Weighted multivariable logistic regression models were used to analyze the relationship between free NPAR and PAD. The nonlinear relationship between NPAR and PAD was investigated using restricted cubic splines. Additionally, subgroup analyses and interaction tests were carried out to provide further understanding.

**Results:**

This study analyzed data from 31,126 NHANES participants (1999–2004), focusing on those aged ≥ 40 years undergoing ABI tests (*n* = 9,970). After exclusions, the final sample was 5,470 participants, representing 79,363,231 U.S. adults. The weighted prevalence of peripheral artery disease (PAD) was 3.75% (95% CI: 3.20–4.38%). Logistic regression analysis revealed significant associations between higher NPAR levels and increased prevalence of PAD. In the adjusted models, the odds ratios (ORs) for the highest versus the lowest NPAR tertile were significant (OR: 1.18, 95% CI: 1.05–1.33). Restricted cubic spline (RCS) analysis showed a positive correlation between NPAR and PAD prevalence (overall *p* = 0.005), though the nonlinear effect was not statistically significant (*p* = 0.504). Stratified analyses indicated significant associations in specific subgroups, such as males (OR: 1.18, 95% CI: 1.10–1.27) and non-diabetics (OR: 1.12, 95% CI: 1.06–1.18), those without CVD (OR: 1.10, 95% CI: 1.04, 1.16), white participants (OR: 1.11, 95% CI: 1.03–1.19), moderate drinkers (OR: 1.14, 95% CI: 1.05–1.25), heavy drinkers (OR:1.19, (95% CI: 1.06–1.33), sedentary individuals (OR:1.10, 95% CI: 1.01–1.19), and former smokers (OR:1.12, 95% CI: 1.04–1.21).

**Conclusion:**

Our study demonstrates a positive correlation between NPAR levels and the prevalence of PAD, implying that elevated NPAR levels are linked to a greater probability of developing PAD. As a cross-sectional study, it cannot establish the temporal sequence of events or causality.

## Introduction

Peripheral arterial disease (PAD) is a chronic condition characterized by atherosclerosis, primarily affecting the lower limbs [1]. It manifests through symptoms such as leg pain or claudication, which result from inadequate blood flow due to the narrowing or blockage of arteries supplying the lower extremities [2]. PAD is a significant global health concern, affecting roughly 10% of the population worldwide, with 15-20% of cases occurring in individuals over 70 years of age [3].

The economic impact and health risks posed by PAD are substantial and should not be underestimated. Therefore, understanding the underlying causes of PAD and implementing effective interventions is essential. Key risk factors for PAD include smoking, diabetes, high cholesterol, hypertension, advancing age, and obesity [4]. Research has established that inflammation, endothelial dysfunction, and oxidative stress are pivotal in the development of PAD [5, 6].

Inflammatory indices such as the neutrophil-to-lymphocyte ratio (NLR), platelet-to-lymphocyte ratio (PLR), and systemic immune-inflammation index (SII) have gained traction in assessing inflammation and disease prognosis. Nonetheless, these indices have limitations. NLR, SII, and PLR rely solely on blood cell counts from a complete blood count (CBC), ignoring the changes in protein metabolism that occur during the inflammatory response [7].

Neutrophils, as vital components of the innate immune system, are closely linked to the incidence and severity of cardiovascular diseases (CVD) [8]. Serum albumin, which plays an important role in transporting substances and regulating osmotic pressure, has been shown to offer protective effects against various cardiovascular conditions. These benefits are attributed to its anti-inflammatory, antioxidant, and antiplatelet properties [9, 10]. The Neutrophil percentage-to-albumin ratio combines neutrophil count, which indicates an acute inflammatory response, with albumin levels, which tend to decrease during chronic inflammation [11]. This ratio serves as a useful marker for evaluating a patient’s inflammatory state, infection, and nutritional health. NPAR has been linked to several cardiovascular diseases [12–14], but its association with PAD risk remains unclear. As PAD tends to worsen over time, identifying reliable markers for early detection of adverse outcomes is crucial. This study aims to investigate the relationship between NPAR levels and PAD prevalence using data from the National Health and Nutrition Examination Survey (NHANES), to identify novel biomarkers for predicting PAD. Through this analysis, we hope to provide a scientific foundation for the early detection and intervention of PAD, offering valuable insights for both future research and clinical practice.

## Methods

### Participants

**Fig. 1:**
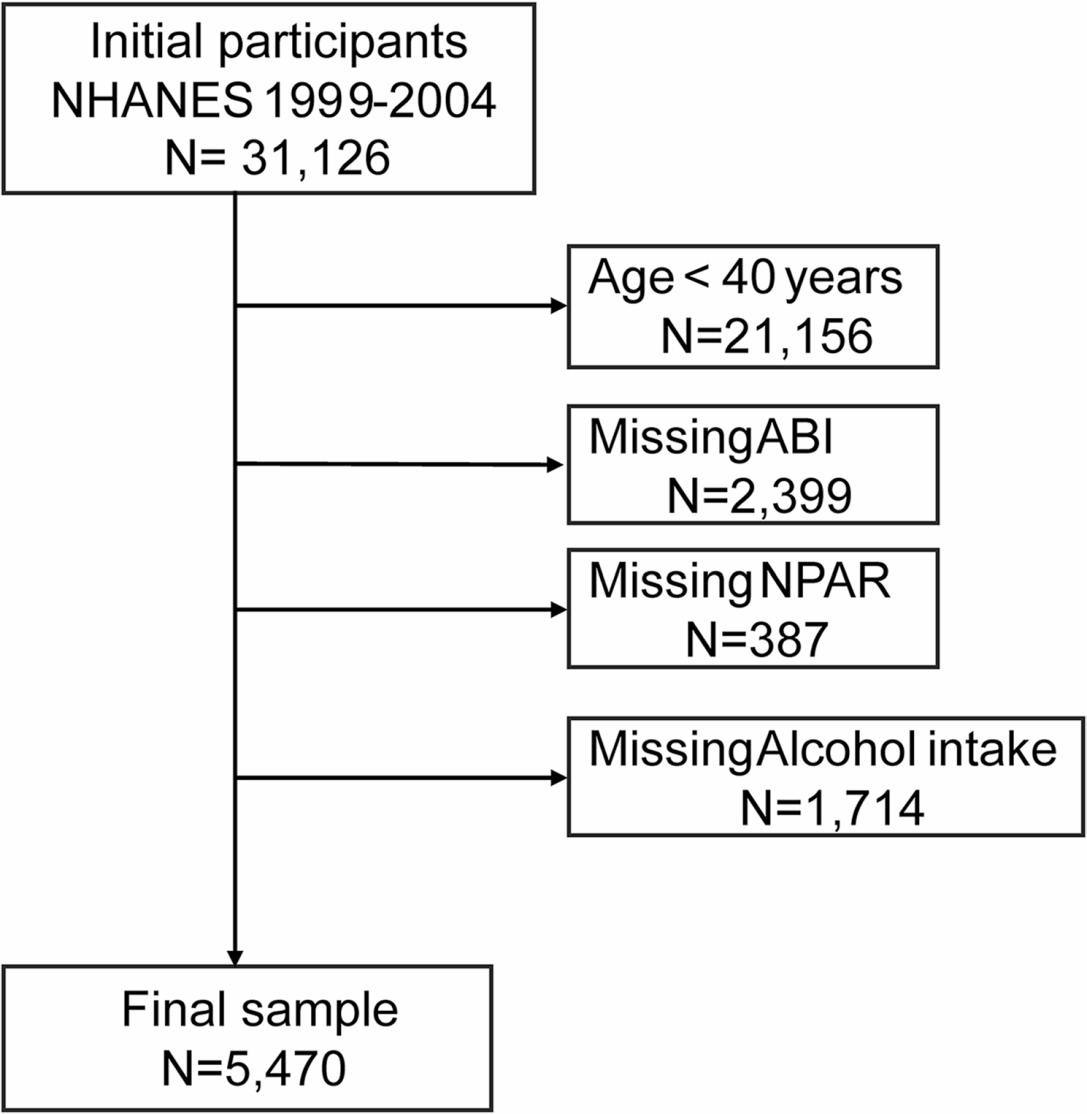
Flowchart of participant selection. NHANES, National Health and Nutrition Examination Survey; NPAR, neutrophil percentage-to-albumin ratio; ABI, ankle-brachial index. Note: No manual alterations were applied to this image

The analysis utilized three cycles of NHANES data, spanning from 1999 to 2004. Initially, we extracted information from a cohort of 31,126 individuals. Following this, we removed cases with incomplete NPAR or Ankle-brachial index (ABI) data, excluded participants under the age of 40, and omitted covariates with a missing data rate exceeding 10%. After implementing these exclusion criteria, the final analytical sample included 5,470 participants. The process of forming the study cohort is depicted in a flowchart (Fig. 1).

## Main and outcome variables

### Ankle-brachial index

The data for this study was obtained from the NHANES database (http://www.cdc.gov/nchs/nhanes.htm*).* The Ankle-brachial index (ABI) was obtained by dividing the mean systolic blood pressure in the ankle by the mean blood pressure in the arm. ABI measurement in NHANES for the years 1999–2002 and 2003–2004 followed a defined protocol. PAD was characterized by an ABI value of less than 0.9 in either leg.

NPAR was determined by the formula: Neutrophil percentage (%) × 100/Albumin (g/dL). We employed tertile cut points to categorize the study population based on the distribution of NPAR and previous research [15]. This approach was adopted considering the skewed distribution of NPAR and the need for meaningful group comparisons to identify potential risk gradients. Tertile cut points for NPAR were determined based on unweighted percentiles. NPAR was analyzed as both a continuous variable and a categorical variable. For the categorical analysis, NPAR was divided into three groups based on tertiles: <12.73, 12.73–14.64, and > 14.64.

## Covariates

The covariates included in this analysis were age, gender, race, education level, poverty-income ratio (PIR), Body Mass Index (BMI), waist circumference (WC), smoking status, physical activity, alcohol consumption, hypertension, diabetes, history of cardiovascular disease (CVD), hypercholesterolemia, estimated glomerular filtration rate (eGFR), and serum homocysteine.

- Age was categorized into < 60 years and ≥ 60 years.
- Education level was classified as less than high school, high school graduate or equivalent (GED), and more than high school.
- The poverty-income ratio was grouped as below the poverty line, at or above the poverty line, and high income.
- Race was classified as Mexican American, non-Hispanic Black, non-Hispanic White, and other.
- Hyperhomocysteinemia was defined as serum homocysteine levels > 15 mg/dL.
- Hypertension was identified if participants had a systolic blood pressure ≥ 140 mmHg and/or diastolic blood pressure ≥ 90 mmHg, or if they self-reported the use of antihypertensive medication or a diagnosis of hypertension.
- Hypercholesterolemia was defined as total serum cholesterol ≥ 240 mg/dL or self-reported use of cholesterol-lowering medication or physician diagnosis.
- Diabetes was identified if HbA1c > 6.5%, fasting blood glucose ≥ 126 mg/dL, self-reported physician diagnosis, or use of oral hypoglycemics or insulin.
- eGFR was calculated using the CKD-EPI equation [16], with values categorized into three groups: ≥90 mL/min/1.73 m², 60–89 mL/min/1.73 m², and < 60 mL/min/1.73 m².
- Smoking status was determined based on self-report, with categories of current, former, or never smokers.
- Alcohol intake was classified as non-drinker, light drinker (1–4 drinks/month), moderate drinker (5–10 drinks/month, or heavy drinker (≥ 10 drinks/month).
- BMI was categorized as underweight (< 18.5 kg/m²), normal (18.5–24.9 kg/m²), overweight (25–29.9 kg/m²), or obese (≥ 30 kg/m²).
- Waist circumference was used as a measure of central adiposity, with high WC defined as > 88 cm for women and > 102 cm for men [17].
- Physical activity was classified as sedentary, light-intensity (daily life activities), or moderate/vigorous (heavy work or physical exertion).
- Participants were considered to have CVD if they reported a history of heart attack, congestive heart failure, coronary heart disease, or angina.

### Statistical analysis

R Studio (version 4.2.1) was used to conduct all statistical tests, with all analyses being weighted. Continuous variables are shown as medians along with interquartile ranges, whereas categorical variables are reported as frequencies and percentages. To examine the risk factors linked to peripheral artery disease, a weighted logistic regression model was employed. We evaluated the presence of multicollinearity among the independent variables using the Variance Inflation Factor (VIF).

### Weighting procedure

To ensure the representativeness of our analysis, we selected the smallest subpopulation weight that encompasses all variables included in our analysis. Specifically, since our analysis involves data from fasting subsamples, we utilized the fasting weights (WTSAF2YR). To account for the complex survey design of NHANES, which includes stratification and clustering, we employed the survey package in R. This package allows us to properly handle the survey design features, ensuring that our estimates and standard errors are accurate.

## Survey design definition

We defined the survey design object using the svydesign function, specifying the stratification variable (SDMVSTRA), the primary sampling unit (PSU) variable (SDMVPSU), and the fasting weights (WTSAF2YR).

For the main analysis, PAD was the outcome variable, and a weighted multivariate logistic regression model was used. Three models were constructed:

- Non-adjusted model: No variables were adjusted.
- Model I: Adjusted for age, race, and gender.
- Model II: Adjusted for age, gender, race, smoking status, alcohol intake, CVD, physical activity, diabetes, and hypertension.

Subgroup analyses were performed based on age, gender, race, smoking status, alcohol intake, CVD, physical activity, diabetes, and hypertension. Odds ratios (OR) with 95% confidence intervals (CI) were used to report effect sizes. Additionally, to assess the non-linear relationship between NPAR and PAD, we used restricted cubic splines (RCS) with knots positioned at the 5th, 35th, 65th, and 95th percentiles of NPAR. The non-linearity test was performed using a likelihood ratio test comparing the RCS model with a linear model. Missing data for variables with less than 10% missing values were imputed using the multiple imputation approach, utilizing the “mice” R package. We generated 10 imputed datasets. The imputation methods were chosen based on the variable type: predictive mean matching for continuous variables, logistic regression for binary variables, and polytomous regression for categorical variables. Convergence diagnostics were enabled to assess the stability of the imputation process.

## Results

### Baseline characteristics

A total of 31,126 individuals participated in the NHANES study from 1999 to 2004, and those aged 40 or older were selected to undergo an ankle-brachial index (ABI) test (*n* = 9,970). Participants with incomplete bilateral ABI data (*n* = 2,399) or non-NPAR (*n* = 387) were excluded. Additionally, since the missing data for alcohol intake exceeded 10%, the missing data were removed (*n* = 1,714). The mean NPAR value in the PAD group exceeded that of the non-PAD group. Furthermore, the distribution of patients with varying NPAR levels differed between the PAD and non-PAD groups. Detailed information can be found in Table 1. After applying weights, this sample represents a population of 79,363,231 adults in the United States (Supplementary materials 1). The overall weighted prevalence of PAD in the study population was 3.75% (95% CI: 3.20 − 4.38%). The weighted prevalence of PAD was calculated for each tertile as follows: <12.73: 2.08% (95% CI: 1.37 − 2.79%),12.74–14.64: 3.97% (95% CI: 3.01 − 4.93%), > 14.64: 5.26% (95% CI: 4.02 − 6.50%).

**Table 1:** Baseline characteristics of the study population

Characteristics	Overall (*n* = 5470)	Without PAD (*n* = 5155)	With PAD (*n* = 315)	*P*-value
Age median [IQR] (year)	59.00 (48.00-69.75)	57.00 (47.00–68.00)	72.00 (64.00–81.00)	< 0.01
NPAR median [IQR]	13.64 (12.18–15.16)	13.57 (12.14–15.10)	14.56 (13.21–16.20)	< 0.01
Sex (%)				0.42
Male	2934 (53.64%)	2772 (53.77%)	162 (51.43%)	
Female	2536 (46.36%)	2383 (46.23%)	153 (48.57%)	
Race (%)				0.08
Mexican American	1293 (23.64%)	1236 (23.98%)	57 (18.10%)	
White	3130 (57.22%)	2939 (57.01%)	191 (60.63%)	
Black	899 (16.44%)	839 (16.28%)	60 (19.05%)	
Other	148 (2.71%)	141 (2.74%)	7 (2.22%)	
Education level (%)				< 0.01
Less than High School	1622 (29.65%)	1496 (29.02%)	126 (40.00%)	
High School Graduate/GED	1296 (23.69%)	1221 (23.69%)	75 (23.81%)	
More Than High School	2552 (46.65%)	2438 (47.29%)	114 (36.19%)	
PIR (%)				< 0.01
Below Poverty Line	678 (12.39%)	629 (12.20%)	49 (15.56%)	
At or Above Poverty Line	3577 (65.39%)	3350 (64.99%)	227 (72.06%)	
High Income	1215 (22.21%)	1176 (22.81%)	39 (12.38%)	
BMI (%)				0.99
Underweight	56 (1.02%)	53 (1.03%)	3 (0.95%)	
Normal	1507 (27.55%)	1421 (27.57%)	86 (27.30%)	
Overweight	2207 (40.35%)	2081 (40.37%)	126 (40.00%)	
Obesity	1700 (31.08%)	1600 (31.04%)	100 (31.75%)	
Waist circumference (%)				< 0.01
Normal	2357 (43.09%)	2249 (43.63%)	108 (34.29%)	
High	3113 (56.91%)	2906 (56.37%)	207 (65.71%)	
Hypertension (%)				< 0.01
No	2598 (47.50%)	2520 (48.88%)	78 (24.76%)	
Yes	2872 (52.50%)	2635 (51.12%)	237 (75.24%)	
CVD (%)				< 0.01
No	4808 (87.90%)	4581 (88.87%)	227 (72.06%)	
Yes	662 (12.10%)	574 (11.13%)	88 (27.94%)	
Smoking status (%)				< 0.01
Never smoker	2339 (42.76%)	2244 (43.53%)	95 (30.16%)	
Current smoker	1200 (21.94%)	1114 (21.61%)	86 (27.30%)	
Former smoker	1931 (35.30%)	1797 (34.86%)	134 (42.54%)	
Physical activity (%)				< 0.01
Light-intensity	1316 (24.06%)	1200 (23.28%)	116 (36.83%)	
Sedentary behaviors	3057 (55.89%)	2897 (56.20%)	160 (50.79%)	
Moderate/vigorous	1097 (20.05%)	1058 (20.52%)	39 (12.38%)	
Alcohol intake (%)				< 0.01
Non-drinker	797 (14.57%)	728 (14.12%)	69 (21.90%)	
Light-drinker	955 (17.46%)	904 (17.54%)	51 (16.19%)	
Moderate-drinker	2588 (47.31%)	2445 (47.43%)	143 (45.40%)	
High-drinker	1130 (20.66%)	1078 (20.91%)	52 (16.51%)	
Hypercholesterolemia (%)				0.99
No	4427 (80.93%)	4172 (80.93%)	255 (80.95%)	
Yes	1043 (19.07%)	983 (19.07%)	60 (19.05%)	
HCY (%)				< 0.01
No	5107 (93.36%)	4836 (93.81%)	271 (86.03%)	
Yes	363 (6.64%)	319 (6.19%)	44 (13.97%)	
eGFR (%)				< 0.01
< 60	571 (10.44%)	487 (9.45%)	84 (26.67%)	
60–89	2208 (40.37%)	2055 (39.86%)	153 (48.57%)	
≥ 90	2691 (49.20%)	2613 (50.69%)	78 (24.76%)	
Diabetes (%)				< 0.01
No	4575 (83.64%)	4352 (84.42%)	223 (70.79%)	
Yes	895 (16.36%)	803 (15.58%)	92 (29.21%)	
NPAR tertile				< 0.01
< 12.73	1822 (33.31%)	1760 (34.14%)	62 (19.68%)	
12.74–14.64	1821 (33.29%)	1717 (33.31%)	104 (33.02%)	
> 14.64	1827 (33.40%)	1678 (32.55%)	149 (47.30%)	
Age categorical (%)				< 0.01
< 60	2774 (50.71%)	2727 (52.90%)	47 (14.92%)	
≥ 60	2696 (49.29%)	2428 (47.10%)	268 (85.08%)	

Data are presented as median [IQR] or *n* (%). IQR, interquartile range

*Abbreviations PAD* Peripheral Arterial Disease, *BMI* body mass index, *PIR*poverty-income ratio, *PIR* was classified as Below Poverty Line,At or Above Poverty Line, High Income, Below Poverty Line: PIR value is less than 1,At or Above Poverty Line: PIR value is greater than 1 and less than 5, High Income: PIR value greater than or equal to 5.00, *HCY* hyperhomocysteinemia, *CVD* cardiovascular diseases, *eGFR* estimated glomerular filtration rate, *NPAR* neutrophil percentage‐to‐albumin ratio: high Waist circumference defined as >88 cm for women and >102 cm for men. Missing data for variables with less than 10% missing values were imputed. Missing data were handled using multiple imputation with the mice package in R

### Association between the NPAR and the prevalence of PAD

The weighted univariable logistic regression model identified several potential covariates linked to PAD prevalence, including age, gender, race, educational level, poverty-to-income ratio, physical activity, smoking habits, alcohol consumption, waist circumference, hypertension, CVD, hypercholesterolemia, hyperhomocysteinemia, eGFR, and diabetes. Multivariate logistic regression results suggested that factors such as race, age, gender, hypertension, smoking status, alcohol intake, CVD, physical activity, and diabetes were associated with PAD prevalence (supplementary materials 2).

As illustrated in Table 2, treating NPAR as a continuous variable revealed that higher NPAR values were linked to an increased prevalence of PAD in the unadjusted model. This relationship remained significant in both Models I and II. When NPAR was categorized into tertiles, with the first tertile serving as the reference group, the unadjusted model revealed a positive correlation between higher NPAR levels and the prevalence of PAD. In Model I, adjusting for race, age, and gender, elevated NPAR values were still correlated with a greater prevalence of PAD. In Model II, after controlling for additional confounding variables such as hypertension, smoking, alcohol use, CVD, physical activity, and diabetes, higher NPAR was found to be an independent risk factor for PAD, relative to lower NPAR.

**Table 2:** Association between the NPAR and the risk of PAD

Variables	Non-adjusted model		Model I		Model II	
	OR (95%CI)	*P* value	OR (95%CI)	*P* value	OR (95%CI)	*P* value
NPAR, ml/g	1.18 (1.12, 1.25)	< 0.0001	1.13 (1.07, 1.20)	0.0002	1.10 (1.04, 1.17)	0.0027
NPAR(Tertiles), ml/g						
< 12.73	Reference		Reference		Reference	
12.74–14.64	1.95 (1.24, 3.05)	0.006	1.79 (1.10, 2.91)	0.0249	1.80 (1.10, 2.94)	0.0262
> 14.64	2.60 (1.88, 3.60)	< 0.0001	2.01 (1.42, 2.85)	0.0004	1.81 (1.27, 2.58)	0.0026
* p* trend		< 0.0001		0.0006		0.0048

Data are presented as the estimated value with its 95% confidence interval

Non-adjusted model: No variables were adjusted

Model I: Adjusted for age, race, and gender

Model II: Adjusted for age, gender, race, smoking status, alcohol intake, CVD, physical activity, diabetes, and hypertension

*Abbreviations* *NPAR* Neutrophil-Percentage-to-Albumin Ratio, *CVD* cardiovascular diseases, *OR* odds ratio, *CI* confidence intervals

### Nonlinearity analysis using RCS

**Fig. 2:**
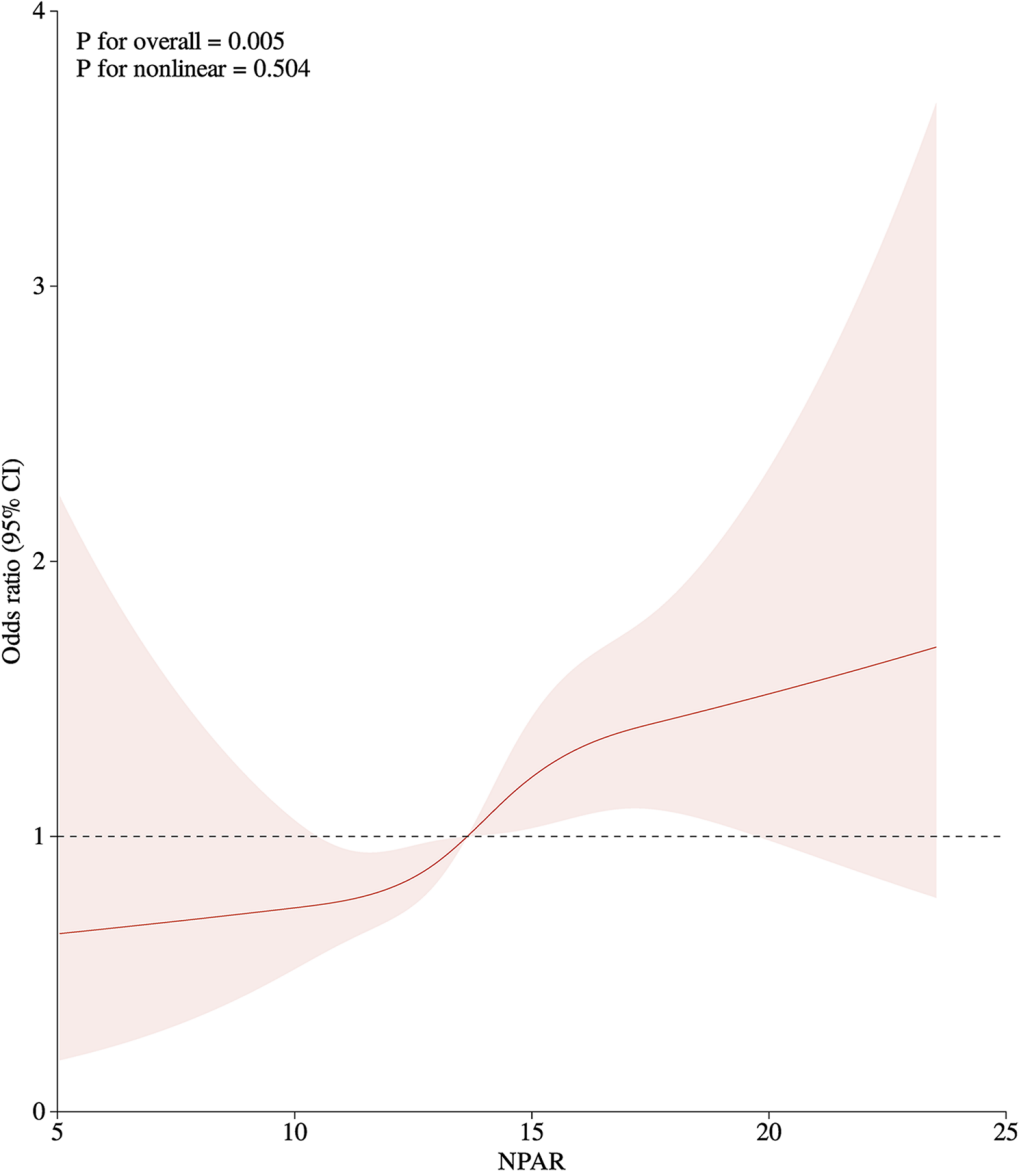
Restricted cubic spline fitting of the relationship between NPAR and peripheral arterial disease (the spline analysis adjusts for age, gender, race, smoking status, alcohol intake, CVD, physical activity, diabetes, and hypertension). The red curve illustrates how the Odds Ratio changes with different levels of NPAR. The pink-shaded area around the red curve represents the 95% CI. The dashed horizontal line at the value of 1 serves as a reference point. An OR of 1 indicates no association between NPAR and the outcome of interest. NPAR, Neutrophil-Percentage-to-Albumin Ratio; CI, confidence intervals. Note: No manual alterations were applied to this image

As illustrated in Fig. 2, the RCS demonstrated a notable positive correlation between NPAR and PAD, with a steady increase observed(overall *p*-value = 0.005). Although the curve shows a certain degree of nonlinear trend, the P-value for the nonlinear effect (0.504) suggests that this nonlinear relationship is not statistically significant.

### Stratification analysis

**Fig. 3:**
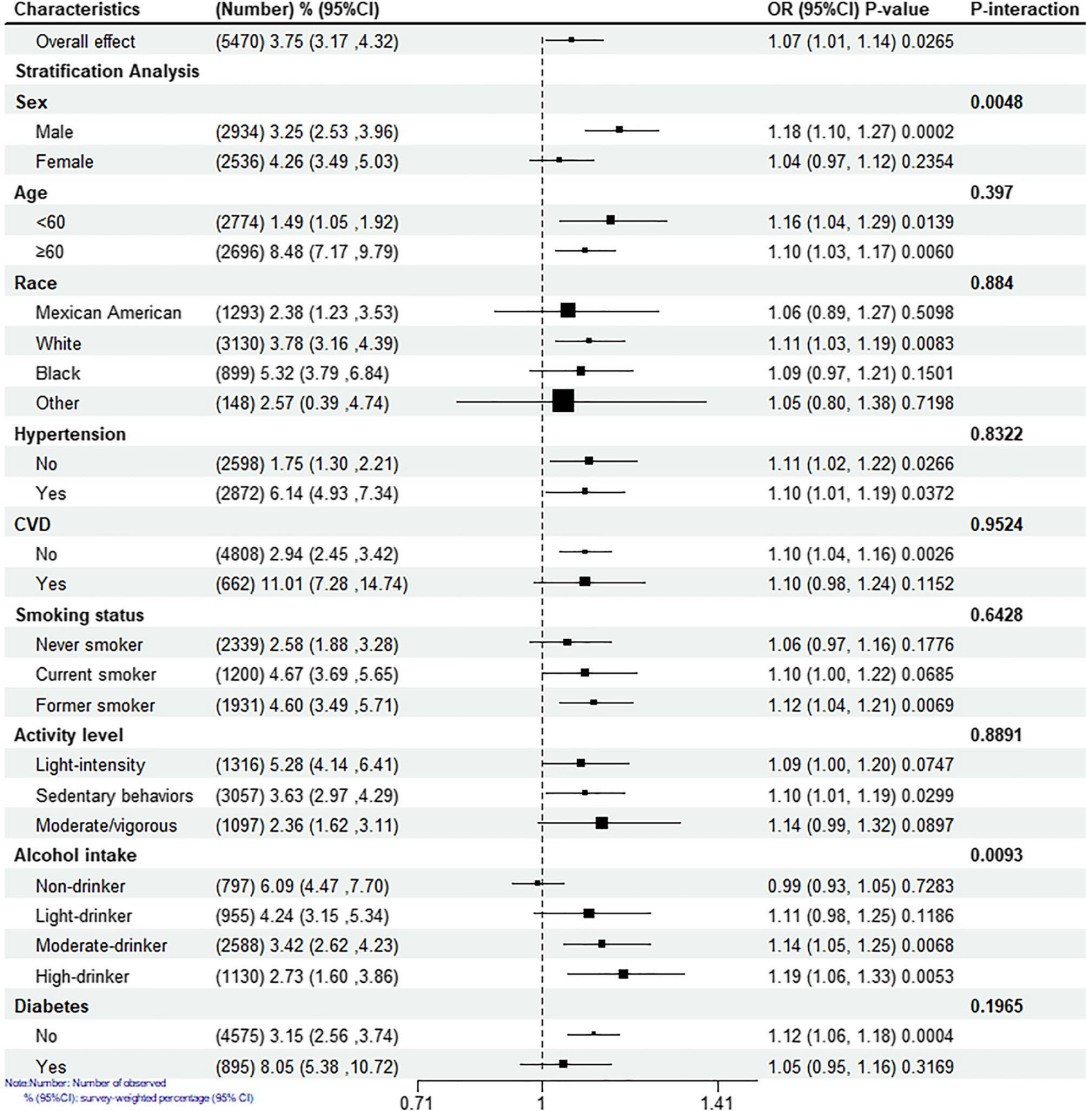
Subgroup analysis and interaction test of NPAR and peripheral arterial disease. The weighting was applied to account for the complex survey design, and the adjustment sets included variables such as race, age, gender, hypertension, smoking status, alcohol intake, CVD, physical activity, and diabetes. CVD, cardiovascular diseases; NPAR, neutrophil percentage-to-albumin ratio; OR, odds ratio; CI, confidence intervals. Note: No manual alterations were applied to this image

Subgroup analyses were performed based on factors such as race, age, gender, hypertension, smoking status, alcohol intake, CVD, physical activity, and diabetes. The forest plot illustrates significant positive associations between NPAR and PAD, particularly in specific subgroups (Fig. 3). These include males (OR: 1.18, 95% CI: 1.10–1.27, *p* = 0.0002), participants without diabetes (OR: 1.12, 95% CI: 1.06–1.18, *p* = 0.0004), those without CVD (OR: 1.10, 95% CI: 1.04, 1.16, *p* = 0.0026), white participants (OR: 1.11, 95% CI: 1.03–1.19, *p* = 0.0083), moderate drinkers (OR: 1.14, 95% CI: 1.05–1.25, *p* = 0.0068), heavy drinkers (OR:1.19, (95% CI: 1.06–1.33,*p* = 0.0053), sedentary individuals (OR:1.10, 95% CI: 1.01–1.19 *p* = 0.0299), and former smokers (OR:1.12, 95% CI: 1.04–1.21, *p* = 0.0069). Additionally, the association between NPAR and PAD was notably stronger in males (p for interaction = 0.0048), as well as in moderate- and heavy-drinking populations (p for interaction = 0.0093).

## Discussion

Studies have shown that NLR and PLR are closely associated with the severity, prognosis, and complications (such as amputation risk) of PAD. The inflammatory state and serum albumin levels are closely related [18–20]. Serum albumin is also an inhibitor of platelet aggregation and low levels of it increase blood viscosity [21]. NPAR combines the neutrophil percentage and albumin level, addressing the limitations of traditional indicators that neglect protein metabolism. NPAR provides a more comprehensive assessment. However, to date, no studies directly examining the relationship between NPAR and PAD have been reported and there are no studies directly comparing the strength of the association of NPAR with that of NLR and PLR in PAD. More research is needed on PAD patients to further clarify the clinical value of NPAR.

This research investigated the relationship between NPAR and PAD. The findings revealed a strong positive association between NPAR levels and the prevalence of PAD. To visualize this connection, a restricted cubic spline model was applied. Furthermore, the stratified analysis confirmed a consistent positive correlation between NPAR and PAD across different population subgroups, supporting the main finding observed in the overall cohort. Notably, subgroup analysis highlighted a significantly elevated PAD prevalence among males and individuals with higher alcohol consumption.

Peripheral arterial disease (PAD) is a chronic condition linked to atherosclerosis, a process where inflammation plays a central role. In atherosclerosis, immune cells such as monocytes and white blood cells, along with cytokines and phospholipids, contribute to the inflammatory cascade [22, 23]. Previous studies have shown that neutrophils, a key type of white blood cell, are significantly involved in PAD [24]. These neutrophils release reactive oxygen species, myeloperoxidase, and neutrophil extracellular traps, which are implicated in the inflammatory response [25–27]. These pro-inflammatory activities contribute to oxidative stress and endothelial dysfunction, leading to the progression of atherosclerosis and increasing the risk of cardiovascular diseases (CVD) [24, 26]. Some studies indicated a notable relationship between albumin levels and inflammation [9, 28]. Albumin has both anti-inflammatory and antioxidant properties, and lower serum albumin levels are associated with higher inflammation, a primary factor in impaired vascular function [29].

The progression of inflammation leads to opposing trends in neutrophils and albumin, making their ratio (NPAR) a valuable indicator of the body’s inflammatory status. NPAR is a novel biomarker derived from routine blood tests, which combine albumin levels and neutrophil percentages. It is easy to measure and repeat, making it a practical tool for assessing cardiovascular risk. Compared to the individual components, NPAR offers a synergistic amplification effect, enhancing its predictive capacity for cardiovascular disease risk [14]. Despite this, there has been limited research examining the relationship between NPAR and PAD.

The restricted cubic spline (RCS) analysis revealed that higher NPAR values are linked to increased PAD prevalence. Although the curve shows a certain degree of nonlinear trend, the non-significant *P*-value for non-linearity (*P* = 0.504) suggests that the relationship can be adequately captured by a linear model. This implies that, within the range of NPAR values observed in our study, the effect on the odds ratio does not deviate significantly from linearity. The non-significant non-linearity test result may be due to the sample size and the distribution of NPAR values in our dataset might not have provided sufficient power to detect a non-linear association if one exists. Future research with larger sample sizes could further explore the potential for non-linear effects.

Subgroup analyses revealed associations that were largely in agreement with the primary findings. The positive relationship between NPAR and PAD was consistent across the population, with variations in race, diabetes status, cardiovascular disease (CVD) status, physical activity, and smoking, although some subgroups did not show statistically significant differences. Although some subgroups did not show statistical significance, we observed notably higher odds ratios (ORs) for PAD prevalence among male participants and those with moderate or high alcohol consumption. Females tend to have lower inflammation levels in chronic diseases [30], and previous studies have suggested that females may exhibit a more rapid resolution of local inflammatory responses, which could mitigate the damaging effects of systemic inflammation on the vasculature [31]. This suggests that the influence of NPAR on PAD prevalence might be attenuated in females, a hypothesis that requires validation in larger studies targeting specific populations.

Some studies have indicated that light-to-moderate alcohol intake may be linked to a lower risk of coronary heart disease [32], heart failure [33], and PAD [34]. Moderate alcohol intake may reduce inflammation through inflammatory markers, potentially lowering the risk of peripheral atherosclerosis and thrombosis [35]. However, recent evidence has revealed a positive linear relationship between alcohol intake and cardiovascular risk, contradicting the idea that alcohol offers cardiovascular benefits at any consumption level [36–38]. Our findings align with this view. Thus, no amount of alcohol appears to confer protection against cardiovascular disease, and excessive drinking can have detrimental effects, especially in heavy drinkers. We propose that alcohol consumption may elevate inflammation and oxidative stress, contributing to endothelial dysfunction and an increased PAD risk. Future research is needed to explore this hypothesis further.

Previous studies have demonstrated that patients with severe hepatic dysfunction, liver cirrhosis, and bacterial infections exhibit elevated NPAR levels [39–41]. Consequently, the existence of infections and liver disease might influence the relationship between NPAR and the prognosis of inflammation. Therefore, it is necessary to conduct stratified analysis in future studies.

### Strength and limitation

This study has several strengths. Firstly, it employed multi-stage complex sampling, ensuring good representativeness of the sample. Secondly, the restricted cubic spline method, combined with stratified analysis, provided robust and reliable results. Thirdly, since neutrophil percentage and albumin levels are routinely measured in complete blood counts, they do not require additional costs, making NPAR a simple and cost-effective tool for identifying PAD risk.

However, there are some limitations to consider. As a cross-sectional study, it cannot establish the temporal sequence of events or causality. Data on disease history and other variables were gathered via questionnaires, which may be subject to recall bias. Additionally, due to NHANES limitations, more comprehensive treatment data and other pertinent factors were either missing or incomplete. Moreover, neutrophil and albumin levels were based on a single measurement, which does not capture dynamic changes in NPAR. Single-time blood measures and non-compressible arteries in diabetes may affect the accuracy of ABI. Although we controlled for numerous potential confounders, many residual confounders remain, such as liver disease, infection, and anthropometric factors. Further investigation is necessary in future research. Lastly, the study’s findings are primarily applicable to US residents, and caution is warranted when attempting to extrapolate these results to populations with differing demographic profiles.

## Conclusion

In conclusion, our results show a positive relationship between NPAR and the prevalence of PAD suggesting that NPAR could be a valuable biomarker for identifying individuals at risk for PAD. This has significant implications for primary prevention, particularly in populations with a high incidence of PAD. Nonetheless, these results should be confirmed in prospective studies to further validate NPAR as a predictive tool for PAD prevalence.

## Data Availability

The data used in this study can be obtained upon request from the corresponding author. Due to privacy and ethical concerns, these data are not accessible to the public.
